# How the complex pharmacology of the fentanyls contributes to their lethality

**DOI:** 10.1111/add.14614

**Published:** 2019-04-17

**Authors:** Hannah Gill, Eamonn Kelly, Graeme Henderson

**Affiliations:** ^1^ School of Physiology, Pharmacology and Neuroscience University of Bristol Bristol UK

**Keywords:** fentanyl, heroin, muscle stiffness, naloxone, overdose, respiratory depression

*Fentanyls as a class of drug have pharmacological properties that make them more likely to lead to overdose than other opioids. In addition to high potency and rapid onset of respiratory depression, these drugs also induce respiratory muscle stiffness, making it more difficult to breathe, are less sensitive than heroin to naloxone reversal and may show reduced cross‐tolerance to other opioids*.

Overdose deaths involving fentanyls (fentanyl and structurally related opioid drugs) have risen dramatically in North America during the last few years 1, while the rest of the world waits nervously to see if this epidemic will spread globally.

Heroin and other opioids depress respiration by acting on μ opioid receptors (MORs) to reduce the response to raised partial pressure of carbon dioxide (pCO_2_) and lowered partial pressure of oxygen (pO_2_), and thus reduce the drive to breathe. Given the high death rate involving fentanyl overdose the question arises of whether the lethality of fentanyls results simply from their high potency or whether other pharmacological properties also contribute.

Fentanyls are relatively selective and potent agonists at MORs, with fentanyl and carfentanil being, respectively, ~×50 and ~×10 000 more potent than morphine 2, 3. Such high potency means that only small amounts of drug are required to produce respiratory depression, and thus even a minor error could result in too much being taken. Fentanyls are highly lipid soluble molecules and readily penetrate into the brain, resulting in overdose levels being reached rapidly. Deaths in heroin overdose may take more than 30 minutes to occur after injection 4, providing a window of opportunity for intervention (administration of the antagonist naloxone). In contrast, fentanyl overdose deaths can occur very quickly, within 5 minutes, potentially before remedial action can be taken 5, 6.

In recent years there has been enormous interest within the pharmacological community in the phenomenon of biased agonism, whereby agonists acting at the same receptor may activate one downstream signalling pathway more strongly over another 7. Activation of MOR results in downstream signalling through either G protein or arrestin pathways. It has been claimed that respiratory depression results from opioid signalling through arrestins 8 and that fentanyl is arrestin‐biased, thus explaining its greater ability to depress respiration than heroin 9. The view that respiratory depression by opioids is mediated by arrestin signalling and that fentanyl is arrestin‐biased is, however, highly contentious. We did not find fentanyl to be arrestin‐biased in our extensive analysis of bias at MOR 10, while in a recent study on transgenic mice, in which the MOR could not signal through arrestins, morphine still depressed respiration 11.

In humans, intravenous administration of fentanyls can produce skeletal muscle rigidity 4, 12, 13. Animal studies show that fentanyls produce muscle rigidity by acting on MORs in various brain regions to enhance motor neurone activity 14. Recently, signs of muscle rigidity have been observed in users at a supervised drug injection facility who have presumably injected illicit fentanyls 15. Skeletal muscle rigidity produces stiffness of the chest wall, referred to colloquially as ‘wooden’ chest. Someone injecting illicit fentanyls may therefore experience the double whammy of a depressed respiratory drive and a mechanical restriction to breathing. Muscle rigidity may also reduce the effectiveness of cardiopulmonary resuscitation. Another consequence of muscle rigidity is that, unlike the characteristic slouching that occurs in heroin overdose, in fentanyl overdose the subject can remain upright and those nearby may be unaware that an overdose has occurred, and where help is summoned those attending may not initially associate body rigidity with opioid overdose 15.

There is increasing awareness that respiratory depression by fentanyls is harder to reverse with naloxone than that by other opioids such as heroin, and may require multiple or higher doses of naloxone 16, 17, 18. Why this should be is unclear, and has mistakenly been attributed to fentanyls having high affinity of binding at MOR. It is a fundamental tenet of pharmacology that under equilibrium‐competitive conditions the degree of antagonism observed depends solely upon the antagonist's affinity and concentration and is independent of the agonist's affinity of binding; i.e. agonists of different affinity should be reversed equally. That fentanyls are less readily reversed may indicate that, due to their high lipid solubility, they have atypical micro‐pharmacokinetic profiles that enhance the agonist:antagonist concentration ratio in the vicinity of the receptors, thus preventing equilibrium competition from taking place, or that they interact with MOR in a novel way that reduces the ability of naloxone to displace them.

In treating heroin overdose the aim has been to keep the dose of naloxone low, administering enough to reverse respiratory depression without precipitating withdrawal symptoms. Further doses of naloxone can be administered if respiration fails to recover. When fentanyl overdose is suspected it may be expedient to rapidly increase the dose of naloxone to reverse the respiratory depression 16.

Tolerance develops to the respiratory depressant effects of opioids. Cross‐tolerance develops between opioid drugs acting at the same receptor, but the degree of cross‐tolerance will depend upon agonist intrinsic efficacy. In a rodent model measuring opioid‐induced locomotor activity, prolonged treatment with morphine produced a 22‐fold increase in the 50% effective dose (ED_50_) for morphine but only a fourfold increase in the ED_50_ for fentanyl 19. Most of the fentanyls have high intrinsic efficacy (ability to produce a response once bound to the receptor) and so need to occupy only a small proportion of the available receptors to produce that response. This suggests that they are less likely to be affected by the loss of receptor function that underlies heroin tolerance and are able to ‘break through’ heroin‐induced tolerance and produce respiratory depression even in heroin‐tolerant individuals. The low level of cross‐tolerance with heroin may be exacerbated by fentanyls also producing chest wall rigidity. As heroin induces little or no chest wall rigidity it is unlikely that tolerance to this effect will have developed in heroin users.

A combination of factors—potency, rate of onset, bias, muscle rigidity, lower susceptibility to naloxone reversal, lack of cross‐tolerance—may conspire together to make fentanyls such lethal drugs. We would suggest that the dangers of fentanyls will only be fully understood and then mitigated by rigorously applying pharmacological principles to both experimental and clinical data.

## Declaration of interests

None.
